# Development of a prototype ChLIA and a research‐use only ELISA for the detection of light chain neurofilament (NfL) in CSF

**DOI:** 10.1002/alz.094720

**Published:** 2025-01-09

**Authors:** Katharina Zeplin, Julia Niewand, Stephanie Franco, Viola Borchardt‐Lohölter, Victor Herbst

**Affiliations:** ^1^ Institute for Experimental Immunology, affiliated to EUROIMMUN Medizinische Labordiagnostika AG, Luebeck, Schleswig‐Holstein Germany; ^2^ EUROIMMUN US Inc, Mountain Lakes, NJ USA

## Abstract

**Background:**

Increased levels of the light chain of neurofilament (NfL) in human cerebrospinal fluid indicate axonal damage. Their determination can support laboratory diagnosis and monitoring of neurological diseases associated with axonal damage. We developed a prototype chemiluminescence assay (ChLIA) and an enzyme‐linked immunoassay (ELISA, research use only) to detect NfL and report their analytical agreement with an established ELISA.

**Method:**

Agreement between the established NF‐light ELISA (UMANDiagnostics) and the Neurofilament Light (NfL) ELISA (EUROIMMUN) as well as the prototype NfL ChLIA (EUROIMMUN) was determined using 19 internal reference samples containing native NfL, with NfL levels distributed across the measurement range of the NF‐light ELISA. Possible bias between methods was estimated with Passing‐Bablok regression and Kolmogorov‐Smirnov CUSUM test.

**Result:**

Comparison of NF‐light ELISA and Neurofilament Light (NfL) ELISA indicated a small proportional deviation with NF‐light ELISA values being higher than Neurofilament Light (NfL) ELISA values (regression: y = ‐63.9+0.97x, 95% CI intercept: [‐490.8, ‐79.67], CI slope: [0.89, 1.05], r = 0.08, CUSUM test p = 0.6). Comparison of Neurofilament Light (NfL) ELISA and prototype NfL ChLIA indicated a small shift with ChLIA values being higher than ELISA values, especially in the upper end of the measurement range (regression: y = 13.73+1.14x, CI intercept: [‐140.9, 97.05], CI slope: [1.07, 1.23], r = 0.98, CUSUM test p = 0.6).

**Conclusion:**

The results of analytical agreement between NfL levels determined by the ELISA and the prototype ChLIA in comparison to the established ELISA show promise regarding their performance characteristics. Being limited by the small sample size, these results will be followed up by a larger method comparison and validation study.

